# Missed Opportunities for Measles, Mumps, and Rubella (MMR) Immunization in Mesoamerica: Potential Impact on Coverage and Days at Risk

**DOI:** 10.1371/journal.pone.0139680

**Published:** 2015-10-27

**Authors:** Ali H. Mokdad, Marielle C. Gagnier, K. Ellicott Colson, Emily Dansereau, Paola Zúñiga-Brenes, Diego Ríos-Zertuche, Annie Haakenstad, Casey K. Johanns, Erin B. Palmisano, Bernardo Hernandez, Emma Iriarte

**Affiliations:** 1 Institute for Health Metrics and Evaluation, 2301 5th Ave, Suite 600, Seattle, Washington, United States of America; 2 Division of Epidemiology, School of Public Health, University of California Berkeley, Berkeley, California, United States of America; 3 Salud Mesoamérica 2015 / Inter-American Development Bank, Calle 50, Edificio Tower Financial Center (Towerbank), Piso 23, Panamá, Panamá; Alberta Provincial Laboratory for Public Health/ University of Alberta, CANADA

## Abstract

**Background:**

Recent outbreaks of measles in the Americas have received news and popular attention, noting the importance of vaccination to population health. To estimate the potential increase in immunization coverage and reduction in days at risk if every opportunity to vaccinate a child was used, we analyzed vaccination histories of children 11–59 months of age from large household surveys in Mesoamerica.

**Methods:**

Our study included 22,234 children aged less than 59 months in El Salvador, Guatemala, Honduras, Mexico, Nicaragua, and Panama. Child vaccination cards were used to calculate coverage of measles, mumps, and rubella (MMR) and to compute the number of days lived at risk. A child had a missed opportunity for vaccination if their card indicated a visit for vaccinations at which the child was not caught up to schedule for MMR. A Cox proportional hazards model was used to compute the hazard ratio associated with the reduction in days at risk, accounting for missed opportunities.

**Results:**

El Salvador had the highest proportion of children with a vaccine card (91.2%) while Nicaragua had the lowest (76.5%). Card MMR coverage ranged from 44.6% in Mexico to 79.6% in Honduras while potential coverage accounting for missed opportunities ranged from 70.8% in Nicaragua to 96.4% in El Salvador. Younger children were less likely to have a missed opportunity. In Panama, children from households with higher expenditure were more likely to have a missed opportunity for MMR vaccination compared to the poorest (OR 1.62, 95% CI: 1.06–2.47). In Nicaragua, compared to children of mothers with no education, children of mothers with primary education and secondary education were less likely to have a missed opportunity (OR 0.46, 95% CI: 0.24–0.88 and OR 0.25, 95% CI: 0.096–0.65, respectively). Mean days at risk for MMR ranged from 158 in Panama to 483 in Mexico while potential days at risk ranged from 92 in Panama to 239 in El Salvador.

**Conclusions:**

Our study found high levels of missed opportunities for immunizing children in Mesoamerica. Our findings cause great concern, as they indicate that families are bringing their children to health facilities, but these children are not receiving all appropriate vaccinations during visits. This points to serious problems in current immunization practices and protocols in poor areas in Mesoamerica. Our study calls for programs to ensure that vaccines are available and that health professionals use every opportunity to vaccinate a child.

## Introduction

Measles mortality dropped globally from 733,000 deaths in 2000 to 164,000 in 2008 [1]. An estimated 13.8 million deaths were avoided between 2000 and 2012 because of measles vaccinations [2]. These great gains have led the World Health Organization to target measles for elimination [3,4].

In the Americas, recent outbreaks of measles, which is generally imported from other countries, have attracted media and popular attention [5,6]. Many children are not immunized, leaving them at risk for contracting the disease. Measles vaccinations remain a key public health measure in the fight against infectious disease in the Americas.

In this study, we assess the presence of missed opportunities to vaccinate using a large household survey in Mesoamerica. We estimate the potential increase in immunization coverage and reduction in days at risk for children aged 11–59 months if every opportunity to vaccinate a child was taken advantage of.

## Methods

### Study design and participants

The data presented were collected as part of the baseline evaluation for Salud Mesoamérica 2015 (SM2015), an initiative established to address the health issues faced by the poorest quintile of the population in El Salvador, Guatemala, Honduras, Nicaragua, Belize, Costa Rica, Panama, and Mexico. In target areas of each country, surveys were conducted in households and health facilities. Censuses were conducted within each selected primary sampling unit, a segment of approximately 150 households, to identify households with women aged 15–49 years and children under 5 years old. Among eligible households, a randomly selected subset was chosen for the SM2015 Household Survey.

The household survey had three components. A household questionnaire captured information on assets, wealth, and characteristics of the home. Each woman aged 15–49 years was asked to complete a maternal health questionnaire that captured demographic, health behavior, and reproductive health information. Caregivers were asked to complete a child health questionnaire on health, diet, and vaccination history for children aged 0–59 months. Trained anthropometrists also conducted physical measurements and anemia tests for these children.

SM2015 baseline surveys were conducted from March 1, 2011 to August 31, 2013. The data were collected using computer‐assisted personal interview (CAPI) software by trained interviewers. Data were continuously monitored by the Institute for Health Metrics and Evaluation (IHME). All data were collected after obtaining informed consent. The field surveyors explained the purpose of this study to participants. Then, written informed consent was obtained from all study participants who agreed to participate prior to data collection. The study received approval from the University of Washington's Institutional Review Board (IRB), and in-country Ministry of Health IRBs, including approval for the procedure for obtaining and recording consent. We used Stata 12.1 and Stata 13.1 for the analyses and all estimates were computed using survey weights, unless otherwise noted. Additional details on SM2015 methodology and implementation are available in a capstone publication on SM2015 [7].

### Definitions

The age of each child was captured in months in the census and household survey (we did not receive the birth day of the children for confidentiality). In order to assess vaccination history, we converted months to days by multiplying reported age in months by 30.4 and adding 15.2 days. We wanted to be more conservative rather than assuming the child birth day is at the beginning or end of their age-month. We then added the number of days between the census survey and the household survey, at which up-to-date vaccination information was captured. This value of age in days was used to determine eligibility for vaccines and calculate time at risk.

Measles, mumps, and rubella (MMR) vaccination is required at 12 months of age in all countries in Mesoamerica. An estimation of MMR coverage was calculated using caregiver recall and vaccine card information. A child was considered compliant if they were 13 months of age or older at the time of the household survey, and they had at least one caregiver-reported or card-documented MMR dose. All children younger than 13 months were considered compliant for MMR. Basically, we gave a grace period of one month by not counting children aged less than 13 months at risk for vaccination. An estimate of card-only coverage was also calculated using the same approach above for card and recall. In order to account for vaccine timelines, we considered children who were vaccinated for MMR before age 11.5 months and did not receive another dose thereafter to be non-compliant since the vaccine was given before age 12 months. The required vaccines were similar in all countries with slight differences.

Children without a vaccination card were excluded from estimation of time at risk. A vaccine was counted as valid when given within 15.2 days of the recommended age in months or any time thereafter. MMR vaccination was valid if administered after age 11.5 months and considered as in the recommended interval if administered between 11.5 and 13.5 months. Children who received an early vaccine were considered at risk starting at the end of the recommended age interval. Days at risk were computed as the number of days from the end of the recommended vaccine interval until receipt of a vaccine. For children with no record of receipt of a vaccine or children who were vaccinated too early, days at risk were computed as their age at the time of the interview minus the date when the vaccine was recommended. This provides a conservative estimate of days at risk, because the number of days at risk for children unvaccinated at the time of interview would increase with time.

A child incurred a “missed opportunity” if their vaccination card indicated a vaccination visit after the recommended vaccine date, but MMR vaccine was not administered at that time. We defined potential coverage as the population vaccination coverage if vaccines were given at missed opportunity visits. Potential days at risk are the number of days that a child went without a vaccine, assuming that the child was caught up at his or her next vaccination visit following recommended vaccine age. Basically, we used the date of the receipt of the vaccine when given later or the time of the survey if the vaccine was not given.

The health facility typically visited for vaccination, as reported by the caregiver, was extracted from the survey and matched to health facilities in the SM2015 baseline Health Facility Survey. Facilities were categorized as providing child services based on the availability of MMR vaccine and stock of oral rehydration salts (ORS). Survey-weighted estimates of MMR coverage, according to the vaccine card, were calculated between children attending facilities with and without MMR and ORS in stock.

### Statistical analyses

We used the χ^2^ test of independence to compare children with and without a vaccine card by socio-demographic variables, stratified by country. The χ^2^ test of independence was also used to compare children who were and were not covered for MMR according to their vaccination card, and children with and without missed opportunities for MMR by socio-demographic variables, stratified by country. Survey-weighted logistic regression was used to determine the association of socio-demographic characteristics with a child having a vaccination card, being covered for MMR according to their vaccination card, and having a missed opportunity for MMR vaccination. All logistic regressions were stratified by country.

We used the Cox proportional hazards model to compute the hazard ratio associated with the reduction in days at risk, after accounting for missed opportunities, and to adjust for other known risk factors in two stages (first, with only country fixed effects, child sex, maternal education, and maternal age; second, with additional covariates) [8]. An unadjusted Kaplan-Meier estimation of time to vaccination was also computed. Responses of children who had not been vaccinated at the time of interview were treated as censored. In each model, indicator variables (0,1) were created to designate individual membership in discrete categories of each of the potential risk factors.

Socio-demographic characteristics were considered at the child, mother, and household level. Child characteristics include gender and categorical age in years. Characteristics of the child’s mother include attained education level (no education, primary education, or secondary or higher education), literacy, age in years at the time of the survey, parity, employment status, and marital status. Household characteristics include household size, gender of the head of household, within-country household monthly expenditure quintile, asset index, and urban or rural locality.

## Results

A total of 22,234 children aged 11–59 months in El Salvador, Guatemala, Honduras, Mexico, Nicaragua, and Panama had vaccination information. Of these, 18,868 had vaccination cards observed on the day of the survey. Table 1 compares child, maternal, and household characteristics of children with and without a vaccine card. The distribution of age, education, and wealth characteristics varies significantly between groups. Child age was an important predictor for having a vaccination card in most countries (Table 2). Older children in El Salvador, Honduras, Nicaragua, and Panama were less likely to have a card than younger children. In Honduras, children whose mothers had secondary or more education were less likely to have a vaccine card than uneducated mothers (odds ratio [OR] 0.390, 95% confidence interval [CI]: 0.202–0.755). Female children in Mexico were more likely to have a vaccine card than males (OR 1.311, 95% CI: 1.125–1.528). Conversely, female children were less likely to have a vaccine card in Nicaragua (OR 0.778, 95% CI: 0.611–0.990). Children in El Salvador were less likely to have a vaccine card if the head of household was female (OR 0.663, 95% CI: 0.485–0.907). In El Salvador and Nicaragua, children of mothers older than 20 were more likely to have a vaccine card. Children in households with more assets were more likely to have a vaccine card in El Salvador (OR 3.225, 95% CI: 1.187–8.760) and Nicaragua (OR 6.220, 95% CI: 1.540–25.11). Mother’s marital status and urbanicity were not significantly associated with a child having a vaccine card in any country when adjusting for other factors.

**Table 1:** Descriptive characteristics comparing children with and without a vaccine card (% unless otherwise noted).

			El Salvador			Guatemala			Honduras			Mexico			Nicaragua			Panama		
			Yes	No	p-value	Yes	No	p-value	Yes	No	p-value	Yes	No	p-value	Yes	No	p-value	Yes	No	p-value
Sample size (N) †			3110	347		4311	880		2620	402		5399	902		1734	467		1694	368	
**Child characteristics**	Female		49.0	48.1	0.748	50.1	50.7	0.764	50.1	52.7	0.328	50.4	44.5	**0.001** **	48.7	52.7	0.130	50.1	54.0	0.183
	Age in years	0 years	18.7	11.8	**<0.001** ***	20.1	18	0.055	19.2	11.9	**<0.001** ***	16.2	26.6	**<0.001** ***	22.9	10.3	**<0.001** ***	17.6	10.3	**<0.001** ***
		1 year	22.7	19.9		22.4	20.6		21.9	13.9		22.1	18.4		21.8	17.8		23.2	19.8	
		2 years	21.6	19.9		21.7	21.6		21.2	22.6		19.5	17.7		19.9	19.7		19.7	21.7	
		3 years	19.4	23.9		19.7	20		19.8	23.9		22	17.8		18.2	23.1		21.5	22.8	
		4 years	17.7	24.5		16.1	19.9		17.9	27.6		20.2	19.4		17.2	29.1		18.1	25.3	
**Maternal characteristics**	Attained education	No education	11.1	10.1	0.280	34.7	33.3	**0.004** **	7.9	7.2	**0.001** **	17.1	25.4	**<0.001** ***	11	9.6	**0.099**	14.2	23.7	**<0.001** ***
		Primary education	56	52.8		51.7	48.5		71.9	63.1		52.3	49.6		51	46.4		57.2	49.3	
		Secondary education or higher	32.9	37.1		13.6	18.2		20.2	29.7		30.7	25		38	43.9		28.7	27	
	Literacy	Illiterate	23	22.5	0.848	63.4	58.1	**0.006** **	36.4	38.3	0.538	43.9	52.9	**<0.001** ***	27.6	24.2	0.167	37.6	45.3	**0.013** *
		Literate	77	77.5		36.6	41.9		63.6	61.7		56.1	47.1		72.4	75.8		62.4	54.7	
	Age in years	Age 15–19	9.4	15.6	**0.001** **	9.9	10.2	0.273	9.6	8.2	**0.006** **	8.1	11.2	**0.012** *	11.6	12.3	0.852	10.6	9.4	0.747
		Age 20–34	70	66.9		68.8	71.1		70.4	63.8		72.1	69.5		70.5	70.8		64.7	64.5	
		Age 35–49	20.6	17.6		21.3	18.7		20	28		19.8	19.3		17.9	16.9		24.7	26.1	
	Parity	1 child	29.4	35.2	0.052	20.9	24	**0.042** *	26.3	22.4	0.294	17.1	19.1	0.505	31.3	30.5	0.559	11.7	15.8	0.070
		2–3 children	41.8	41.2		38.2	40.3		42.1	42.8		42.5	41.8		44.1	47.3		41.7	36.4	
		4–5 children	15.5	14.4		21	18.8		17.5	21.4		21.8	20.4		15.7	13.2		26.1	23.7	
		6 + children	13.3	9.2		19.9	16.9		14.1	13.4		18.6	18.6		8.9	8.9		20.5	24.1	
	Marital status	Single	13.2	16.4	**0.002** **	6.8	9.6	**0.029** *	15.4	17.4	0.509	1.5	2.1	0.337	15.8	20.7	0.058	8	7.3	0.390
		Married	36	25.5		37.9	35.7		33	31.1		35.1	32.7		32.4	33		8.3	5.7	
		Union	40.4	46.6		51.1	49.7		48.7	47.4		58	60		47.2	41.1		76.1	78	
		Divorced, separated, widowed, other	10.4	11.4		4.1	5		2.9	4.1		5.4	5.2		4.6	5.3		7.6	9	
	Employment status	Employed and working	9.5	12.7	**0.002** **	3.1	5.6	**<0.001** ***	8.3	14.3	**0.002** **	4.8	8.8	**<0.001** ***	12.8	21.2	**<0.001** ***	5.6	5.7	0.305
		Homemaker	88.2	82.4		94.7	90.4		89.1	82.3		93.9	89.7		84.7	76.3		92	93.3	
		Employed but not working, student, retired, disabled	2.3	4.9		2.2	4		2.7	3.4		1.3	1.5		2.5	2.5		2.4	1	
**Household characteristics**	Expenditure quintile	Quintile 1	20	17.3	**0.013** *	19.8	24.1	**0.014** *	21.4	17.8	**0.001** **	22.5	**21**	0.050	20.3	21.3	**0.005** **	**19.9**	20.4	**0.019** *
		Quintile 2	21.8	16.1		20.1	16.2		20.5	16.6		22	**20.3**		21.5	14.8		**19.4**	25.3	
		Quintile 3	19.6	20.7		19.9	19.3		19.5	16.8		20.7	**19.2**		20.7	20		**18.6**	17.9	
		Quintile 4	19	19.9		19.7	19.3		20	21.9		18.8	**20**		20	20.6		**20.9**	14.4	
		Quintile 5	19.5	25.9		20.5	21.1		18.7	26.9		16	**19.6**		17.4	23.2		**21.3**	22	
	Average asset index		0.37	0.36	**0.020** *	0.23	0.25	**<0.001** ***	0.24	0.26	**0.004** **	0.23	0.22	**0.021** *	0.24	0.23	**0.005** **	0.18	0.17	**0.002** **
	Average household size (N)		3.07	3.03	0.124	6.63	6.42	0.427	5.75	6.01	**0.001** **	6.02	6.04	**0.024** *	5.88	5.87	0.084	9.31	9.33	**<0.001** ***
	Urbanicity	Rural household	73.2	65.4	**0.002** **	87.7	82	**<0.001** ***	84.3	77.9	**0.001** **	66.6	57.3	**<0.001** ***	69.5	62.2	**0.003** **	100	100	N/A
		Urban household	26.8	34.6		12.3	18		15.7	22.1		33.4	42.7		30.5	37.8		0	0	
	Female head of household		0.26	0.35	**0.001** **	0.12	0.15	**0.017** *	0.18	0.23	**0.033** *	0.07	0.08	0.292	0.25	0.32	**0.001** **	0.27	0.21	**0.045** *

† N varies by variable due to missing values.

* p<0.05

** p<0.01

*** p<0.001

**Table 2:** Child, maternal, and household characteristics associated with a child having a vaccine card^†^.

			El Salvador		Guatemala		Honduras		Mexico		Nicaragua		Panama	
			*N = 2968*		*N = 4677*		*N = 2613*		*N = 5840*		*N = 1983*		*N = 1679*	
			OR	CI	OR	CI	OR	CI	OR	CI	OR	CI	OR	CI
**Child characteristics**	Female		1.133	[0.863,1.488]	1.000	[0.839,1.192]	0.812	[0.618,1.066]	**1.311** ***	[1.125,1.528]	**0.778** *	[0.611,0.990]	0.892	[0.634,1.255]
	Age in years	0 years	1.000	(ref)	1.000	(ref)	1.000	(ref)	1.000	(ref)	1.000	(ref)	1.000	(ref)
		1 year	0.727	[0.479,1.103]	1.086	[0.827,1.427]	0.952	[0.555,1.633]	**1.863** ***	[1.429,2.428]	**0.594** *	[0.378,0.932]	**0.492** *	[0.271,0.895]
		2 years	0.700	[0.438,1.116]	0.937	[0.731,1.202]	**0.545** **	[0.361,0.823]	**1.771** ***	[1.327,2.363]	**0.416** ***	[0.259,0.667]	**0.380** ***	[0.222,0.649]
		3 years	**0.556** **	[0.369,0.838]	0.975	[0.724,1.313]	**0.484** **	[0.296,0.792]	**1.944** ***	[1.454,2.600]	**0.348** ***	[0.231,0.525]	**0.389** ***	[0.245,0.618]
		4 years	**0.467** **	[0.278,0.786]	0.775	[0.575,1.044]	**0.348** ***	[0.209,0.582]	**1.680** ***	[1.246,2.265]	**0.262** ***	[0.150,0.458]	**0.305** ***	[0.183,0.508]
**Maternal characteristics**	Attained education	No education	1.000	(ref)	1.000	(ref)	1.000	(ref)	1.000	(ref)	1.000	(ref)	1.000	(ref)
		Primary education	0.935	[0.529,1.652]	1.057	[0.816,1.370]	0.764	[0.426,1.370]	1.283	[0.888,1.853]	1.010	[0.594,1.718]	**2.018** *	[1.025,3.972]
		Secondary educationor higher	1.064	[0.568,1.994]	0.965	[0.611,1.523]	**0.390** **	[0.202,0.755]	1.293	[0.843,1.983]	0.810	[0.383,1.714]	1.645	[0.790,3.422]
	Literate		1.200	[0.812,1.775]	0.923	[0.701,1.217]	1.326	[0.920,1.911]	**1.535** **	[1.159,2.033]	0.947	[0.638,1.406]	1.126	[0.682,1.858]
	Age in years	Age 15–19	1.000	(ref)	1.000	(ref)	1.000	(ref)	1.000	(ref)	1.000	(ref)	1.000	(ref)
		Age 20–34	**1.686** *	[1.064,2.673]	0.859	[0.586,1.260]	1.311	[0.735,2.339]	1.297	[0.936,1.797]	1.528	[0.889,2.625]	0.792	[0.405,1.548]
		Age 35–49	**2.126** *	[1.125,4.018]	0.936	[0.595,1.472]	0.810	[0.445,1.473]	1.410	[0.873,2.276]	1.985	[0.985,3.997]	0.961	[0.407,2.271]
	Parity	1 child	1.000	(ref)	1.000	(ref)	1.000	(ref)	1.000	(ref)	1.000	(ref)	1.000	(ref)
		2–3 children	1.172	[0.810,1.697]	1.042	[0.801,1.356]	0.791	[0.515,1.216]	1.017	[0.752,1.376]	1.005	[0.684,1.478]	**1.803** *	[1.074,3.027]
		4–5 children	1.051	[0.653,1.690]	1.056	[0.747,1.492]	0.698	[0.413,1.182]	1.046	[0.693,1.579]	1.115	[0.657,1.890]	**1.806** *	[1.069,3.050]
		6 + children	1.211	[0.593,2.474]	0.969	[0.619,1.517]	1.002	[0.541,1.857]	1.057	[0.607,1.842]	0.781	[0.392,1.555]	1.288	[0.650,2.552]
	Marital status	Single	1.000	(ref)	1.000	(ref)	1.000	(ref)	1.000	(ref)	1.000	(ref)	1.000	(ref)
		Married	1.088	[0.655,1.807]	1.100	[0.768,1.576]	1.300	[0.761,2.223]	1.457	[0.645,3.292]	0.962	[0.568,1.632]	1.432	[0.515,3.982]
		Union	0.804	[0.495,1.304]	1.085	[0.778,1.513]	0.952	[0.639,1.419]	1.461	[0.654,3.263]	1.219	[0.800,1.857]	0.810	[0.411,1.595]
		Divorced, separated, widowed, other	1.234	[0.714,2.132]	1.002	[0.595,1.687]	1.041	[0.480,2.258]	1.403	[0.582,3.380]	0.940	[0.430,2.057]	0.737	[0.335,1.623]
	Employment status	Employed and working	1.000	(ref)	1.000	(ref)	1.000	(ref)	1.000	(ref)	1.000	(ref)	1.000	(ref)
		Homemaker	1.264	[0.818,1.954]	1.398	[0.920,2.126]	1.322	[0.885,1.975]	**1.609** *	[1.083,2.389]	**1.548** *	[1.021,2.346]	1.169	[0.532,2.567]
		Employed but not working, student, retired, disabled	0.755	[0.355,1.603]	0.842	[0.499,1.422]	1.645	[0.604,4.479]	1.661	[0.622,4.440]	1.252	[0.374,4.195]	3.072	[0.659,14.32]
**Household characteristics**	Expenditure quintile	Quintile 1	1.000	(ref)	1.000	(ref)	1.000	(ref)	1.000	(ref)	1.000	(ref)	1.000	(ref)
		Quintile 2	1.092	[0.684,1.746]	**1.513** *	[1.103,2.076]	1.082	[0.730,1.602]	0.926	[0.642,1.335]	**1.483** *	[1.027,2.142]	0.835	[0.476,1.463]
		Quintile 3	0.763	[0.481,1.211]	1.178	[0.852,1.629]	1.067	[0.706,1.611]	0.897	[0.635,1.268]	1.176	[0.825,1.677]	1.222	[0.744,2.007]
		Quintile 4	0.788	[0.479,1.296]	1.254	[0.857,1.834]	0.870	[0.560,1.352]	0.837	[0.584,1.200]	1.179	[0.757,1.836]	**1.750** *	[1.001,3.059]
		Quintile 5	**0.539** *	[0.332,0.876]	1.243	[0.895,1.725]	0.772	[0.456,1.307]	0.735	[0.475,1.138]	0.757	[0.454,1.262]	1.014	[0.548,1.874]
	Average asset index		**3.225** *	[1.187,8.760]	0.445	[0.161,1.229]	0.971	[0.197,4.774]	3.006	[0.792,11.41]	**6.220** *	[1.540,25.11]	2.849	[0.254,31.92]
	Average household size		1.016	[0.925,1.116]	1.032	[0.991,1.075]	0.975	[0.908,1.047]	1.007	[0.953,1.065]	1.001	[0.938,1.068]	0.998	[0.951,1.048]
	Urban household		1.180	[0.688,2.022]	1.012	[0.623,1.644]	1.023	[0.477,2.191]	1.383	[0.600,3.189]	0.968	[0.449,2.086]	0.791	[0.361,1.732]
	Female head of household		**0.663** *	[0.485,0.907]	0.925	[0.663,1.290]	0.943	[0.644,1.382]	1.013	[0.651,1.574]	0.834	[0.620,1.121]	1.313	[0.888,1.940]

OR: odds ratio. CI: confidence interval.

Exponentiated coefficients; 95% confidence intervals in brackets

* p<0.05

** p<0.01

*** p<0.001

†Models adjusted for all variables in the table

Table 3 shows a comparison of characteristics between children that were and were not covered for MMR according to their vaccine card. Child age, maternal education and employment, and household wealth have significantly different distributions between groups. However, when adjusting for all characteristics with regression, different patterns emerge among countries (Table 4). In El Salvador, children aged 2 to 3 years were less likely to be covered than those aged 1 year, but children aged 4 years were much more likely (OR 2.095, 95% CI: 1.498–2.929) due to a required MMR booster at 48 months. In Guatemala, children of partnered women were more likely to be covered than those who were single. In Mexico, children from larger families were less likely to be covered, controlling for other factors. Children in Nicaragua were less likely to be covered for MMR if their head of household was female (OR 0.668, 95% CI: 0.500–0.893).

**Table 3:** Descriptive characteristics comparing children with and without coverage for MMR at the time of the survey (% unless otherwise noted).

			El Salvador			Guatemala			Honduras			Mexico			Nicaragua			Panama		
			Yes	No	p-value	Yes	No	p-value	Yes	No	p-value	Yes	No	p-value	Yes	No	p-value	Yes	No	p-value
Sample size (N)†			1266	974		2483	1381		1564	790		2043	2850		904	789		1118	544	
**Child characteristics**	Female		48.7	52	0.131	49.5	51.8	0.161	50.3	51.3	0.643	49.7	50.7	0.478	48.9	49.4	0.826	51.9	51.2	0.794
	Age in years	0 years																		
		1 year	21.1	15.3	**<0.001** ***	25.1	22.2	0.164	23.7	21.4	**0.021** *	23.3	23	0.468	26.3	21.3	**0.022** *	25	23.9	0.670
		2 years	22.9	39.2		27.6	29.2		27.8	26.1		23.3	25.2		26.1	25.2		23.9	26.7	
		3 years	21.6	33.8		26	25.6		26.3	24.6		27.9	26.5		24.7	25.1		27.2	25.9	
		4 years	34.4	11.7		21.3	23		22.2	28		25.4	25.3		22.9	28.4		24	23.5	
**Maternal characteristics**	Attained education	No education	10.9	12.6	0.334	37.2	34.2	0.143	9.2	7	**0.001** **	17.4	18.6	0.085	12.1	10.7	**0.009** **	13.8	21.3	**0.001** **
		Primary education	57.4	54.7		49.9	51.4		73.1	68.2		52.3	54		54	47.8		56.6	54.9	
		Secondary education or higher	31.8	32.7		12.9	14.4		17.7	24.8		30.4	27.4		33.9	41.5		29.6	23.8	
	Literacy	Illiterate	23.2	25.5	0.197	65	62.6	0.154	37.3	37.7	0.849	43.3	47.8	**0.002** **	28.8	26.6	0.322	36.5	46.2	**0.001** **
		Literate	76.8	74.5		35	37.4		62.7	62.3		56.7	52.2		71.2	73.4		63.5	53.8	
	Age in years	Age 15–19	7.1	7.7	0.745	7.5	7.3	0.962	6.9	7.1	0.050	6.7	6.4	0.176	9.3	9.4	0.985	8.4	9.2	0.907
		Age 20–34	70.1	68.7		70.4	70.5		72.5	67.6		73.6	71.7		71	71.2		66.1	65.6	
		Age 35–49	22.8	23.6		22	22.2		20.6	25.3		19.7	21.9		19.7	19.4		25.4	25.2	
	Parity	1 child	27.9	26.9	0.756	18.6	18.3	0.454	24.2	23.4	0.185	15.1	14.5	0.141	29.8	29.8	0.732	10.9	13	0.439
		2–3 children	42.2	41.3		38.7	41.2		41.2	45		44.9	42.2		45.1	43.6		42.5	38.6	
		4–5 children	15.3	16.9		22.9	21.1		19	19.3		21	23.3		15.2	17.3		26	26	
		6 + children	14.6	14.9		19.8	19.5		15.6	12.3		18.9	20		9.9	9.4		20.6	22.4	
	Marital status	Single	13.9	11.8		6.1	8.5		13.8	17.5		1.4	1.7		16	17.1		7.7	8.3	
		Married	35.6	39.1		38.7	37.4		33.9	32.6		35.9	34.4		33.1	33.9		8.6	6	
		Union	39.6	38.9	0.271	50.7	49.4	0.064	48.8	46.5	0.194	57.2	58	0.579	46.4	43.9	0.757	75.7	77.5	0.401
		Divorced, separated, widowed, other	10.8	10.1		4.5	4.7		3.5	3.4		5.5	5.9		4.6	5.1		8	8.3	
	Employment status	Employed and working	9.7	10.3	0.267	3.2	4	**0.023** *	7.7	13.5	**<0.001** ***	5.1	6.2	0.192	13.1	18.8	**0.006** **	6.4	4.3	0.203
		Homemaker	88.4	86.8		94.6	92.5		89.6	83.9		93.7	92.4		84.9	78.6		91.1	93.9	
		Employed but not working, student, retired, disabled	1.9	2.9		2.2	3.6		2.7	2.6		1.2	1.4		2	2.6		2.4	1.8	
**Household characteristics**	Expenditure quintile	Quintile 1	19.6	20.8	0.931	19.1	23	**0.043** *	22.3	18.6	**<0.001** ***	23.6	21.7	0.403	22	20.6	**0.006** **	19.6	19.9	**0.008** **
		Quintile 2	22.2	21.4		19.8	17.6		20.2	19.1		21.6	22.4		22.9	17.7		18.3	23.7	
		Quintile 3	19	19.4		20.1	20.6		20.7	17.2		20.3	20		19.3	21.1		19	17.3	
		Quintile 4	19.4	19.5		19.6	18.6		19.7	21		18.9	18.8		20.2	19.2		21.6	15.4	
		Quintile 5	19.7	18.9		21.4	20.2		17.1	24		15.6	17.1		15.6	21.4		21.5	23.7	
	Average asset index		0.37	0.37	0.438	0.23	0.24	0.256	0.24	0.25	**0.015** *	0.23	0.23	0.050	0.23	0.24	0.679	0.18	0.17	**0.002** **
	Average household size (N)		3.04	3.1	0.173	6.61	6.55	0.794	5.74	5.75	**0.012** *	5.97	6.01	0.795	5.64	5.93	0.115	9.16	9.39	0.050
	Urbanicity	Rural household	73.8	72.4	0.460	87.8	84.3	**0.002** **	85.1	80.2	**0.002** **	65	66.4	0.314	71.2	64.6	**0.004** **	100	100	n/a
		Urban household	26.2	27.6		12.2	15.7		14.9	19.8		35	33.6		28.8	35.4		0	0	
	Female head of household		0.27	0.26	0.641	0.13	0.14	0.339	0.18	0.22	**0.022** *	0.08	0.08	0.444	0.24	0.31	**<0.001** ***	0.28	0.22	**0.015** **

† N varies by variable due to missing values.

* p<0.05

** p<0.01

*** p<0.001

**Table 4:** Child, maternal, and household characteristics associated with a child being covered for MMR^†^.

			El Salvador		Guatemala		Honduras		Mexico		Nicaragua		Panama	
			*N = 1950*		*N = 3445*		*N = 2003*		*N = 4510*		*N = 1510*		*N = 1346*	
			OR	CI	OR	CI	OR	CI	OR	CI	OR	CI	OR	CI
**Child characteristics**	Female		0.949	[0.767,1.174]	0.937	[0.802,1.095]	0.938	[0.754,1.167]	0.977	[0.864,1.106]	0.863	[0.713,1.046]	1.045	[0.830,1.314]
	Age in years	0 years	N/A		N/A		N/A		N/A		N/A		N/A	
		1 year	1.000	(ref)	1.000	(ref)	1.000	(ref)	1.000	(ref)	1.000	(ref)	1.000	(ref)
		2 years	**0.446** ***	[0.340,0.585]	0.835	[0.695,1.004]	1.078	[0.826,1.408]	0.928	[0.754,1.143]	0.866	[0.621,1.206]	0.944	[0.633,1.408]
		3 years	**0.471** ***	[0.348,0.637]	0.898	[0.736,1.095]	1.181	[0.849,1.642]	1.065	[0.872,1.302]	0.787	[0.589,1.052]	1.112	[0.804,1.539]
		4 years	**2.095** ***	[1.498,2.929]	0.858	[0.665,1.106]	0.876	[0.630,1.219]	1.033	[0.844,1.264]	**0.594** **	[0.424,0.831]	1.075	[0.745,1.549]
**Maternal characteristics**	Attained education	No education	1.000	(ref)	1.000	(ref)	1.000	(ref)	1.000	(ref)	1.000	(ref)	1.000	(ref)
		Primary education	1.037	[0.699,1.539]	0.834	[0.666,1.044]	0.753	[0.467,1.213]	0.898	[0.709,1.137]	0.943	[0.594,1.497]	1.424	[0.808,2.511]
		Secondary education or higher	0.857	[0.513,1.431]	0.836	[0.568,1.230]	0.590	[0.326,1.068]	0.853	[0.631,1.152]	0.702	[0.381,1.296]	1.605	[0.795,3.240]
	Literate	1.208	[0.850,1.719]	1.021	[0.822,1.269]	1.257	[0.955,1.654]	1.182	[0.938,1.488]	1.096	[0.787,1.526]	1.194	[0.779,1.828]
	Age in years	Age 15–19	1.000	(ref)	1.000	(ref)	1.000	(ref)	1.000	(ref)	1.000	(ref)	1.000	(ref)
		Age 20–34	1.158	[0.746,1.797]	0.871	[0.613,1.239]	1.109	[0.713,1.725]	0.961	[0.685,1.348]	1.352	[0.847,2.159]	0.854	[0.530,1.376]
		Age 35–49	1.060	[0.640,1.754]	0.809	[0.535,1.223]	0.789	[0.471,1.320]	0.977	[0.654,1.458]	1.465	[0.839,2.559]	1.041	[0.578,1.873]
	Parity	1 child	1.000	(ref)	1.000	(ref)	1.000	(ref)	1.000	(ref)	1.000	(ref)	1.000	(ref)
		2–3 children	0.917	[0.686,1.226]	0.853	[0.672,1.083]	0.798	[0.597,1.067]	0.937	[0.769,1.142]	1.099	[0.813,1.484]	1.265	[0.804,1.989]
		4–5 children	0.808	[0.563,1.161]	0.9	[0.677,1.195]	0.85	[0.607,1.191]	**0.734** *	[0.556,0.968]	0.818	[0.561,1.193]	1.138	[0.712,1.819]
		6 + children	0.863	[0.516,1.444]	0.856	[0.602,1.217]	1.158	[0.741,1.810]	**0.679** *	[0.464,0.994]	0.965	[0.555,1.677]	1.067	[0.644,1.769]
	Marital status	Single	1.000	(ref)	1.000	(ref)	1.000	(ref)	1.000	(ref)	1.000	(ref)	1.000	(ref)
		Married	0.728	[0.492,1.077]	**1.433** *	[1.057,1.945]	1.340	[0.965,1.861]	1.698	[0.808,3.569]	0.695	[0.457,1.057]	1.877	[0.890,3.961]
		Union	0.866	[0.588,1.275]	**1.402** *	[1.023,1.923]	1.212	[0.905,1.623]	1.632	[0.758,3.515]	0.769	[0.546,1.082]	1.079	[0.595,1.957]
		Divorced, separated, widowed, other	0.862	[0.571,1.303]	1.408	[0.943,2.103]	1.458	[0.722,2.943]	1.151	[0.546,2.429]	0.825	[0.479,1.421]	0.935	[0.455,1.921]
	Employment status	Employed and working	1.000	(ref)	1.000	(ref)	1.000	(ref)	1.000	(ref)	1.000	(ref)	1.000	(ref)
		Homemaker	0.988	[0.675,1.447]	1.031	[0.654,1.625]	**1.452** *	[1.032,2.043]	1.171	[0.814,1.686]	1.402	[0.908,2.164]	0.922	[0.446,1.908]
		Employed but not working, student, retired, disabled	0.787	[0.419,1.478]	0.633	[0.365,1.100]	1.944	[0.905,4.173]	0.845	[0.359,1.992]	1.484	[0.619,3.559]	1.399	[0.441,4.435]
**Household characteristics**	Expenditure quintile	Quintile 1	1.000	(ref)	1.000	(ref)	1.000	(ref)	1.000	(ref)	1.000	(ref)	1.000	(ref)
		Quintile 2	1.115	[0.837,1.486]	**1.356** *	[1.027,1.790]	1.016	[0.726,1.423]	0.797	[0.608,1.044]	1.364	[0.912,2.040]	0.839	[0.515,1.366]
		Quintile 3	1.093	[0.797,1.501]	1.110	[0.844,1.459]	1.081	[0.727,1.608]	0.839	[0.646,1.089]	0.958	[0.676,1.358]	1.168	[0.783,1.742]
		Quintile 4	1.236	[0.923,1.655]	1.281	[0.945,1.736]	0.856	[0.568,1.293]	0.811	[0.603,1.091]	1.312	[0.866,1.989]	1.305	[0.816,2.086]
		Quintile 5	1.259	[0.885,1.791]	1.201	[0.891,1.618]	0.702	[0.458,1.076]	0.842	[0.612,1.160]	0.907	[0.569,1.444]	0.868	[0.535,1.407]
	Average asset index		0.894	[0.466,1.713]	0.613	[0.299,1.255]	0.710	[0.243,2.076]	0.818	[0.246,2.719]	1.557	[0.493,4.922]	3.759	[0.732,19.30]
	Average household size		0.989	[0.910,1.074]	1.007	[0.975,1.041]	1.010	[0.958,1.065]	1.047	[0.994,1.103]	0.961	[0.904,1.023]	0.988	[0.954,1.024]
	Urban household		0.808	[0.618,1.056]	0.747	[0.516,1.080]	0.828	[0.589,1.164]	1.028	[0.716,1.476]	0.914	[0.626,1.335]	N/A		
	Female head of household		1.069	[0.802,1.424]	1.005	[0.763,1.325]	0.869	[0.660,1.145]	1.134	[0.800,1.607]	**0.668** **	[0.500,0.893]	1.341	[0.995,1.808]

OR: odds ratio. CI: confidence interval.

Exponentiated coefficients; 95% confidence intervals in brackets

†Models adjusted for all variables in the table

* p<0.05

** p<0.01

*** p<0.001

The coverage cascade of MMR immunization by country is shown in Table 5. El Salvador had the highest proportion of children with a vaccine card (91.2%) while Nicaragua had the lowest (76.5%). Countries with low card coverage had more dramatic differences between coverage according to card and recall versus card-only coverage. When considering the timing of MMR vaccines, children in Mexico had an average of 483 days at risk after age 13.5 months; this was higher than the average in Panama (158 days) and Guatemala (187 days). If children were “caught up” with the vaccination scheme, the potential coverage of MMR in Panama could be 97.1% as compared to the 77.3% observed.

**Table 5:** Coverage cascade of MMR by country.

Country	Number of children aged 0–59 months	Proportion owning health card (%)	MMR coverage according to card and recall (%) *	MMR coverage according to card only (%) * †	MMR coverage according to card only considering timeliness (%) * ‡	Mean days at risk	Total person-days at risk	Potential card-only MMR coverage (%) *	Mean potential days at risk	Total potential person-days at risk
El Salvador	3,457	91.2	90.9	77.3	56.8	347	779,251	96.4	239	410,256
Guatemala	5,191	83.0	84.9	77.5	64.0	187	601,468	80.7	148	433,871
Honduras	3,022	86.7	93.8	79.6	66.9	217	451,916	83.2	152	290,203
Mexico	6,301	85.0	73.5	44.6	38.7	483	2,031,114	85.6	185	315,540
Nicaragua	2,201	76.5	90.7	66.1	52.9	241	322,216	70.8	170	209,967
Panama	2,062	81.0	88.1	72.0	66.2	158	219,270	78.9	92	92,895

* Coverage for children 13.5–59 months.

† Excluding children without vaccination cards. If the child has completed the number of required doses for age, they are considered compliant.

‡ Excluding children without vaccination cards. If the child has completed the number of required doses for age and not before the eligibility window, they are considered compliant.

Table 6 shows the distribution of child, maternal, and household characteristics between children with and without a missed opportunity for MMR vaccination, by country. Younger age groups were less likely to have a missed opportunity. After adjustment for potential confounders, younger children remained less likely to have a missed opportunity. In Panama, children from households in higher expenditure quintiles were more likely to have a missed opportunity than those from the poorest (OR 1.623, 95% CI: 1.064–2.475), accounting for other factors (Table 7). In Nicaragua, compared to children of mothers with no education, children of mothers with primary education and secondary education were less likely to have a missed opportunity (OR 0.458, 95% CI: 0.239–0.878 and OR 0.251, 95% CI: 0.0965–0.652, respectively) (Table 7). In El Salvador, children of partnered women were more likely to have a missed opportunity than those with single mothers.

**Table 6:** Descriptive characteristics comparing children with and without missed opportunities for MMR vaccine (% unless otherwise noted).

			El Salvador			Guatemala			Honduras			Mexico			Nicaragua			Panama		
			Yes	No	p-value	Yes	No	p-value	Yes	No	p-value	Yes	No	p-value	Yes	No	p-value	Yes	No	p-value
Sample size (N)†			644	1596		394	2889		300	1738		2727	1621		216	1077		450	901	
**Child characteristics**	Female		51.9	49.4	0.298	52.8	50	0.295	53.7	49.9	0.233	51.3	50.1	0.443	54.6	47.7	0.064	51.8	50.4	0.630
	Age in years	0 years	n/a			n/a			n/a			n/a			n/a			n/a		
		1 year	19.1	18.4	**<0.001** ***	9.4	26.6	**<0.001** ***	6.7	27.3	**<0.001** ***	20.2	28.5	**<0.001** ***	13.4	28.3	**<0.001** ***	23.8	26.7	**0.002** **
		2 years	26.1	31.6		26.4	28.7		26	27.5		23.9	24.8		19	28.2		20.7	26.6	
		3 years	20.8	29.4		30.5	25.3		30.3	24.6		28.8	24.9		29.2	23.5		27.1	26.9	
		4 years	34	20.7		33.8	19.4		37	20.6		27	21.8		38.4	20		28.4	19.8	
**Maternal characteristics**	Attained education	No education	12.1	11.4	0.499	43.7	35.6	**0.011** *	11.2	8.2	0.189	18.2	15.9	**0.008** **	20.7	10.4	**<0.001** ***	15.4	14.1	0.826
		Primary education	54.3	57		46.2	51.6		72.3	72.5		54.1	52.2		50.8	52		57.1	57.5	
		Secondary education or higher	33.6	31.6		10.1	12.8		16.5	19.3		27.6	31.9		28.5	37.6		27.5	28.4	
	Literacy	Illiterate	25.6	23.7	0.334	69.9	64.6	0.055	38.1	37.6	0.867	47.6	41	**<0.001** ***	39.4	26.7	**<0.001** ***	40	37	0.326
		Literate	74.4	76.3		30.1	35.4		61.9	62.4		52.4	59		60.6	73.3		60	63	
	Age in years	Age 15–19	6.8	7.6	0.083	6.7	7.1	0.968	5.6	7	0.699	5.7	6.8	**0.037** *	8.8	9.4	0.298	8.9	8.9	0.493
		Age 20–34	66.9	70.5		70.6	70.1		73.4	71.9		72.1	74.2		66.8	71.2		68.4	65.3	
		Age 35–49	26.2	21.9		22.7	22.8		21	21.1		22.1	19.1		24.4	19.4		22.7	25.9	
	Parity	1 child	28.8	27	0.311	15.1	18.2	0.108	18.6	24.9	0.132	13.5	15.9	0.100	29.3	30.3	0.533	10.9	10.6	0.296
		2–3 children	40	42.5		38.7	39.2		46.8	41.9		43.4	43.9		40.8	44.3		45.5	40.8	
		4–5 children	14.8	16.4		27.5	22.1		20.5	18.4		23.4	21.4		19.9	15.7		26.1	26.6	
		6 + children	16.4	14.1		18.8	20.5		14.1	14.8		19.7	18.7		9.9	9.7		17.6	21.9	
	Marital status	Single	11	13.8	0.050	5.6	6.5	0.272	13.9	14.6	0.484	1.5	1.4	0.456	18.7	15.1	0.473	8.6	7.9	0.809
		Married	41.3	35.5		39.2	38.4		31.5	34.7		34.5	36.8		33.2	34.1		8.9	7.9	
		Union	37.7	40		48.7	50.8		52.1	47.2		58.1	56.5		45.1	45.9		73.9	76.5	
		Divorced, separated, widowed, other	10	10.8		6.4	4.3		2.6	3.5		5.9	5.3		3.1	4.9		8.6	7.7	
	Employment status	Employed and working	10.8	9.6	0.235	2.5	3.2	0.758	7.1	9.1	0.387	5.2	4.9	0.903	13	14.3	0.871	6.1	5.7	0.773
		Homemaker	87.7	87.7		94.9	94.6		89.5	88.6		93.5	93.9		84.5	83.4		91.1	92.1	
		Employed but not working, student, retired, disabled	1.6	2.6		2.5	2.2		3.4	2.4		1.3	1.3		2.6	2.3		2.9	2.2	
**Household characteristics**	Expenditure quintile	Quintile 1	21	19.8	0.717	21	19.5	0.786	19.7	21.9	0.121	23.2	22.3	0.608	23.6	20.7	0.516	16	21.2	0.060
		Quintile 2	21.1	22.1		19	19.9		21	20.6		22.9	21.5		20.4	22.4		18.4	19.5	
		Quintile 3	20.7	18.6		19.7	20.7		25	18.8		20	20.8		17.1	20.8		21.3	17.2	
		Quintile 4	18.8	19.7		17.7	19.3		17	20.4		18.7	18.9		19.4	19.6		23.3	19.9	
		Quintile 5	18.5	19.7		22.6	20.7		17.3	18.3		15.2	16.6		19.4	16.5		20.9	22.2	
	Average asset index		0.37	0.37	0.973	0.22	0.23	0.781	0.24	0.24	0.127	0.23	0.24	0.050	0.23	0.24	0.154	0.17	0.18	**0.022** *
	Average household size (N)		3.12	3.04	0.086	7.1	6.54	**0.002** **	5.71	5.7	0.374	6.01	5.96	0.329	5.97	5.7	0.075	9.26	9.14	0.451
	Urbanicity	Rural household	74.1	72.8	0.542	86.7	88.1	0.413	85.7	84.3	0.547	69.3	64.1	**<0.001** ***	68.5	70.2	0.616	100	100	N/A
		Urban household	25.9	27.2		13.3	11.9		14.3	15.7		30.7	35.9		31.5	29.8		0	0	
	Female head of household		0.25	0.26	0.635	0.14	0.13	0.358	0.17	0.18	0.655	0.07	0.08	0.623	0.3	0.24	0.095	0.24	0.28	0.168

† N varies by variable due to missing values.

* p<0.05

** p<0.01

*** p<0.001

**Table 7:** Child, maternal, and household characteristics associated with a child having a missed opportunity for MMR vaccine^†^.

			El Salvador		Guatemala		Honduras		Mexico		Nicaragua		Panama	
			*N = 1950*		*N = 2970*		*N = 1786*		*N = 4021*		*N = 1179*		*N = 1100*	
			OR	CI	OR	CI	OR	CI	OR	CI	OR	CI	OR	CI
**Child characteristics**	Female		1.140	[0.911,1.425]	1.164	[0.878,1.542]	1.027	[0.778,1.356]	1.030	[0.884,1.201]	1.383	[0.965,1.980]	1.032	[0.757,1.406]
	Age in years	0 years	N/A		N/A		N/A		N/A		N/A		N/A	
		1 year	1.000	(ref)	1.000	(ref)	1.000	(ref)	1.000	(ref)	1.000	(ref)	1.000	(ref)
		2 years	0.782	[0.590,1.036]	**2.929** ***	[1.978,4.336]	**4.681** ***	[2.417,9.066]	**1.366** **	[1.127,1.655]	1.382	[0.753,2.538]	0.886	[0.577,1.360]
		3 years	**0.679** *	[0.469,0.982]	**3.829** ***	[2.510,5.842]	**5.967** ***	[3.427,10.39]	**1.694** ***	[1.387,2.070]	**3.456** ***	[2.045,5.839]	**1.193**	[0.740,1.923]
			**1.617** **	[1.152,2.269]	**5.462** ***	[3.524,8.467]	**8.816** ***	[4.939,15.74]	**1.830** ***	[1.467,2.283]	**5.006** ***	[3.108,8.062]	**1.705** **	[1.176,2.470]
**Maternal characteristics**	Attained education	No education	1.000	(ref)	1.000	(ref)	1.000	(ref)	1.000	(ref)	1.000	(ref)	1.000	(ref)
		Primary education	1.059	[0.722,1.554]	**0.728** *	[0.538,0.987]	0.710	[0.436,1.155]	1.088	[0.861,1.375]	**0.458** *	[0.239,0.878]	0.956	[0.672,1.361]
		Secondary education or higher	1.306	[0.800,2.130]	0.693	[0.421,1.141]	0.641	[0.333,1.236]	1.055	[0.777,1.432]	**0.251** **	[0.0965,0.652]	0.896	[0.497,1.614]
	Literate		0.796	[0.565,1.122]	0.937	[0.661,1.329]	1.183	[0.833,1.680]	0.857	[0.681,1.077]	0.794	[0.504,1.253]	0.907	[0.623,1.321]
	Age in years	Age 15–19	1.000	(ref)	1.000	(ref)	1.000	(ref)	1.000	(ref)	1.000	(ref)	1.000	(ref)
		Age 20–34	1.001	[0.646,1.553]	0.726	[0.427,1.233]	0.908	[0.473,1.743]	1.130	[0.815,1.566]	0.892	[0.508,1.567]	0.920	[0.577,1.469]
		Age 35–49	1.376	[0.763,2.480]	0.575	[0.293,1.125]	0.831	[0.386,1.791]	1.303	[0.862,1.969]	1.081	[0.493,2.370]	0.808	[0.456,1.434]
	Parity	1 child	1.000	(ref)	1.000	(ref)	1.000	(ref)	1.000	(ref)	1.000	(ref)	1.000	(ref)
		2–3 children	**0.695** *	[0.526,0.917]	0.959	[0.670,1.374]	1.295	[0.857,1.958]	1.1	[0.852,1.420]	0.794	[0.484,1.304]	1.016	[0.620,1.665]
		4–5 children	**0.597** **	[0.406,0.880]	1.047	[0.688,1.593]	1.117	[0.687,1.815]	1.108	[0.792,1.552]	0.627	[0.333,1.182]	0.894	[0.481,1.659]
		6+ children	**0.572** *	[0.346,0.946]	0.636	[0.394,1.026]	0.944	[0.472,1.887]	1.184	[0.719,1.951]	**0.389** *	[0.162,0.935]	0.824	[0.418,1.626]
	Marital status	Single	1.000	(ref)	1.000	(ref)	1.000	(ref)	1.000	(ref)	1.000	(ref)	1.000	(ref)
		Married	**1.916** **	[1.248,2.941]	1.065	[0.591,1.920]	0.855	[0.534,1.369]	0.673	[0.354,1.281]	0.723	[0.338,1.548]	1.088	[0.531,2.228]
		Union	**1.493** *	[1.023,2.179]	0.949	[0.511,1.762]	1.087	[0.708,1.667]	0.707	[0.375,1.332]	0.895	[0.502,1.598]	0.861	[0.544,1.364]
		Divorced, separated, widowed, other	1.372	[0.882,2.134]	1.319	[0.619,2.812]	0.791	[0.270,2.321]	0.846	[0.413,1.733]	0.698	[0.228,2.133]	1.040	[0.487,2.221]
	Employment status	Employed and working	1.000	(ref)	1.000	(ref)	1.000	(ref)	1.000	(ref)	1.000	(ref)	1.000	(ref)
		Homemaker	0.916	[0.654,1.283]	1.352	[0.652,2.807]	1.240	[0.754,2.041]	0.847	[0.539,1.329]	1.296	[0.747,2.249]	1.121	[0.618,2.033]
		Employed but not working, student, retired, disabled	**0.420** *	[0.205,0.863]	1.451	[0.502,4.200]	1.847	[0.681,5.006]	1.014	[0.353,2.918]	1.136	[0.345,3.744]	1.498	[0.542,4.140]
**Household characteristics**	Expenditure quintile	Quintile 1	1.000	(ref)	1.000	(ref)	1.000	(ref)	1.000	(ref)	1.000	(ref)	1.000	(ref)
		Quintile 2	0.861	[0.608,1.219]	0.938	[0.641,1.373]	1.231	[0.820,1.847]	1.077	[0.820,1.416]	0.724	[0.376,1.394]	1.441	[0.969,2.143]
		Quintile 3	1.011	[0.692,1.479]	0.959	[0.648,1.420]	**1.546** *	[1.010,2.367]	1.009	[0.779,1.306]	0.815	[0.442,1.501]	**2.054** **	[1.328,3.177]
		Quintile 4	0.879	[0.610,1.265]	0.871	[0.561,1.350]	0.814	[0.507,1.305]	1.066	[0.812,1.398]	0.894	[0.492,1.625]	**1.836** **	[1.231,2.740]
		Quintile 5	0.842	[0.564,1.259]	1.258	[0.832,1.902]	1.168	[0.737,1.852]	0.969	[0.679,1.385]	1.444	[0.714,2.923]	**1.623** *	[1.064,2.475]
	Average asset index		0.857	[0.433,1.693]	0.609	[0.164,2.264]	0.796	[0.113,5.587]	0.718	[0.223,2.309]	0.640	[0.145,2.823]	0.340	[0.0839,1.381]
	Average household size		1.026	[0.945,1.114]	**1.058** *	[1.014,1.105]	1.003	[0.916,1.097]	0.951	[0.890,1.017]	1.064	[0.991,1.143]	1.007	[0.970,1.045]
	Urban household		1.076	[0.763,1.519]	1.336	[0.813,2.197]	0.842	[0.525,1.349]	0.934	[0.655,1.331]	1.523	[0.765,3.033]	N/A
	Female head of household		1.011	[0.745,1.370]	0.992	[0.696,1.415]	0.996	[0.635,1.562]	0.958	[0.668,1.374]	1.162	[0.627,2.155]	0.842	[0.606,1.169]

OR: odds ratio. CI: confidence interval.

Exponentiated coefficients; 95% confidence intervals in brackets

†Models adjusted for all variables in the table

* p<0.05

** p<0.01

*** p<0.001

**Fig 1:**
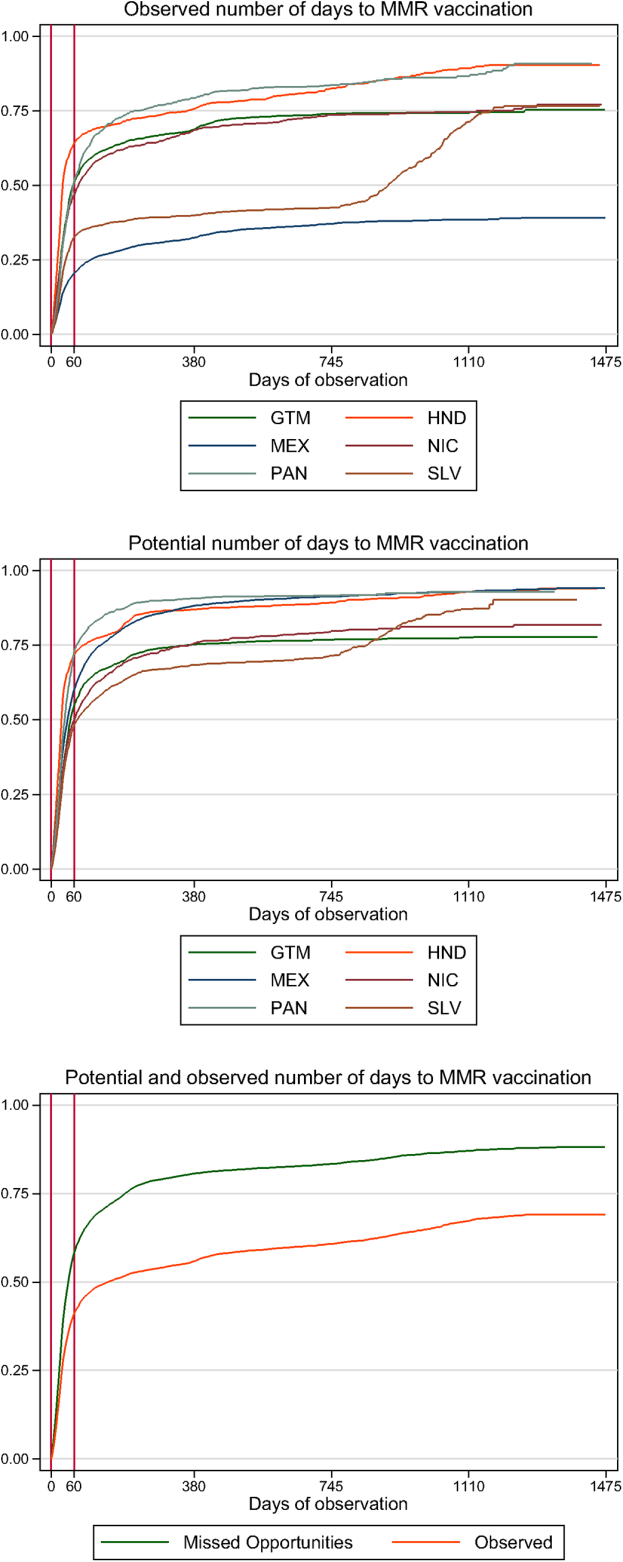
Days to MMR vaccination: observed time to vaccination and potential time to vaccination given missed opportunities by country and pooled*. *Adjusted Kaplan-Meier estimation of the time to MMR vaccination among children with a vaccination card. Covariates are adjusted to the median value across the entire sample; results represent female children age 2 years whose mothers have primary education and are literate, are age 20–34 years with 2–3 children, are homemakers, and are living in a household in the third household expenditure quintile with 23.8% of assets, a male head of household, a household size of five, in a rural area. Vertical red lines indicate the time window in which MMR was considered on time (11.5–13.5 months of age). Labeled days of observation represent the following time points: 0 days = first day that a child is eligible for MMR vaccination (11.5 months of age); 60 days = end of MMR eligibility window (13.5 months); 380 days = age 2 years; 745 days = age 3 years; 1110 days = age 4 years; 1475 days = age 5 years, the oldest age at which a child was included in the sample.

Fig 1 shows the observed days to MMR vaccination compared to potential days to vaccination if children were caught up, between countries and among all countries. The greatest increase in vaccination over time occurred during the recommended vaccine interval; vaccination rates level off after children reach 2 years. Differentiation between observed and potential days to MMR vaccination was largely gained before the child is 2 years. The days at risk could be most reduced in Mexico if missed opportunities were avoided.

Cox proportional hazards models for MMR vaccination are shown in Table 8. Country fixed effects remain significant in all models. Education was not significantly associated with days to MMR vaccination. MMR coverage of children attending health facilities by stocks of MMR and ORS are presented in Table 9. When categorized in this way, MMR coverage did not vary in most countries. The exception was Mexico, where MMR coverage among children attending facilities with ORS (46.2%) was significantly higher than those without ORS in stock (22.5%).

**Table 8:** Cox proportional hazard model for MMR coverage^†^.

			Model 1		Model 2	
			*N = 13311*		*N = 13006*	
			Hazard ratio	CI	Hazard ratio	CI
**Model 1 covariates**	Missed Opportunity		**2.468** ***	[2.232,2.729]	**2.470** ***	[2.232,2.733]
	Country	Guatemala	1.000	(ref)	1.000	(ref)
		Honduras	1.042	[0.947,1.147]	1.035	[0.938,1.142]
		Mexico	0.669***	[0.620,0.723]	**0.673** ***	[0.619,0.731]
		Nicaragua	0.829***	[0.746,0.921]	**0.822** ***	[0.739,0.915]
		Panama	1.222***	[1.114,1.339]	**1.254** ***	[1.125,1.398]
		El Salvador	0.678***	[0.619,0.744]	**0.640** ***	[0.568,0.721]
	Female		0.985	[0.940,1.031]	0.986	[0.942,1.032]
	Attained education	No education	1.000	(ref)	1.000	(ref)
		Primary education	1.032	[0.969,1.099]	0.995	[0.928,1.067]
		Secondary education or higher	1.023	[0.945,1.107]	0.970	[0.884,1.063]
	Age in years	Age 15–19	1.000	(ref)	1.000	(ref)
		Age 20–34	0.963	[0.856,1.084]	1.016	[0.899,1.148]
		Age 35–49	0.908	[0.799,1.033]	0.997	[0.861,1.154]
**Child characteristics**	Age in years	0 years			N/A	
		1 year			1.000	(ref)
		2 years			**0.836** ***	[0.778,0.898]
		3 years			**0.820** ***	[0.762,0.883]
		4 years			**0.809** ***	[0.748,0.875]
**Maternal characteristics**	Literate				1.077*	[1.011,1.146]
	Parity	1 child			1.000	(ref)
		2–3 children			0.992	[0.919,1.070]
		4–5 children			0.944	[0.855,1.042]
		6 + children			1.005	[0.882,1.145]
	Marital status	Single			1.000	(ref)
		Married			0.945	[0.813,1.099]
		Union			0.957	[0.831,1.102]
		Divorced, separated, widowed, other			0.988	[0.849,1.148]
	Employment status	Employed and working			1.000	(ref)
		Homemaker			1.122	[0.983,1.281]
		Employed but not working, student, retired, disabled			1.122	[0.876,1.438]
**Household characteristics**	Expenditure quintile	Quintile 1			1.000	(ref)
		Quintile 2			0.975	[0.899,1.057]
		Quintile 3			0.942	[0.854,1.039]
		Quintile 4			0.998	[0.916,1.088]
		Quintile 5			0.930	[0.838,1.032]
	Average asset index				1.110	[0.827,1.491]
	Average household size				0.987	[0.971,1.003]
	Urban household				0.943	[0.860,1.033]
	Female head of household				0.983	[0.891,1.086]

CI: confidence interval.

95% confidence intervals in brackets

†Models adjusted for all variables indicated in the column

* p<0.05

*** p<0.001

**Table 9:** Estimates of MMR coverage among children attending health facilities based on MMR stock and ORS stock*.

		Stock of MMR				Provision of oral rehydration salt (ORS)		
		Number of facilities	MMR coverage among children attending facilities with MMR in stock on day of survey [95% CI]	MMR coverage among children attending facilities with MMR out of stock on day of survey [95% CI]	MMR coverage among children attending facilities with MMR stock-out in three months prior to the survey† [95% CI]	Number of facilities	MMR coverage among children attending facilities with ORS in stock on day of survey [95% CI]	MMR coverage among children attending facilities with ORS out of stock on day of survey [95% CI]
Guatemala	N	42	1237	4	74	61	1433	132
	%		64.4% [60.6–68.2%]	73.4% [0–100%]	71.1% [64.0–78.3%]		64.7% [61.1–68.3%]	66.5% [54.9–78.2%]
Honduras	N	55	826	127	0	58	900	76
	%		69.0% [64.1–73.9%]	72.2% [66.3–782%]	-		69.3% [64.7–74.0%]	73.6% [61.3–85.9%]
Mexico	N	23	555	139	75	28	605	196
	%		34.5 [25.1–44.0%]	58.6% [32.0–85.1%]	50.1% [25.9–74.3%]		46.2% [34.3–58.1%]	22.5% [11.5–33.4%]
Nicaragua	N	19	425	34	0	25	384	87
	%		45.5% [38.5–52.5%]	60.8% [43.9–77.7%]	-		48.2% [40.1–56.3%]	44.2% [32.2–56.1%]
Panama	N	17	711	37	32	30	706	239
	%		72% [67.4–76.6%]	72.3% [59.3–85.3%]	71.7% [31.5–100%]		71.6% [65.7–77.5%]	74.7% [68.2–81.3%]

MMR: measles, mumps, rubella vaccine. ORS: oral rehydration salts.

*Excluding children without health cards. If the child has completed the number of required doses for age with proper time interval and not before the eligibility window, they are considered compliant. Children are matched to health facilities based on caregiver-reported usual location for vaccination as matched to the baseline measurement of the SM2015 Health Facility Survey. El Salvador is not included because usual facility for vaccination was not ascertained.

†Among health facilities that had MMR in stock on the day of the survey.

When we examined what vaccines were given during a visit with a MMR missed opportunity, the patterns varied by country. Oral polio vaccine had the highest percentage of vaccines given when MMR was not in El Salvador (46.7%), Guatemala (56.1%), and Nicaragua (44.4%). While hepatitis A accounted for 48.9% in Panama, pneumococcal for 32.9% in Mexico, and diphtheria, pertussis, and tetanus (DPT) for 41.0% in Honduras (S3 Table). Moreover, MMR coverage would have increased by 38.5%, 30%, 24.2%, and 25.4% if no missed opportunity occurred in year 1, 2, 3, and 4, respectively.

## Discussion

Our study, based on large surveys in poor areas of Mesoamerica, exposed high levels of missed opportunities to immunize children. Our finding is concerning, as it indicates that families are bringing their children to health facilities, but serious problems in current immunization practices and protocols exist in poor areas in Mesoamerica. Our study emphasizes the need for programs to ensure that vaccines are available and that health professionals use every opportunity to vaccinate a child.

Missed opportunities to vaccinate children during health care visits or the failure to administer needed vaccines during a visit lead to lower national vaccination coverage levels. Using all clinical encounters to screen for needed vaccines and, when indicated, to immunize has been recommended by medical and public health experts since 1992 [9]. However, one limitation of our study is that we calculated missed opportunities using only vaccination visits, as data on other health care visits were not available. Moreover, we do not have data on whether a mother or other relative brought a child to a clinic during health visits for the parent or a sibling. Using all visits as possibilities for vaccination would result in increased coverage in children and subsequently less morbidity and mortality.

The findings we report could be explained in a number of ways. First, missed opportunities could be a result of a shortage of vaccines; the MMR vaccine may simply not have been available on the day of the visit. Our findings call for more attention to the shortage of vaccines at health facilities. However, country-level shortages and problems with supplies and logistics are tractable, manageable challenges. Measures to request vaccines periodically and safety mechanisms to request supplies when stocks are low, can ensure availability. The business model of supply and demand may also be a means to improve logistics. Through SM2015, countries may learn from each other by discussing these challenges and success stories across Mesoamerica.

Second, reluctance to administer immunizations when a child is ill, failure to immunize at all well-child care visits, and inadequate knowledge of current immunization schedules may also be major reasons for missed opportunities to immunize [10–14]. Efforts to decrease missed opportunities that focus on changing physician knowledge have had variable success. Siegel et al. showed that, despite reporting a good knowledge of contraindications to immunization, physicians were still reluctant to administer vaccines at acute care visits when vaccination was not contraindicated [12]. Reminders have been shown to be effective at changing physician behavior. Szilagyi et al. showed that screening by nurses at the time of the visit and attaching reminder cards to the chart increased the rate of vaccine administration by providers [14]. Computer-generated reminder systems can improve the performance of practicing physicians. The most common and effective type of reminder system is a patient-specific report, which is made available to the physician at the time of an encounter. Automatic computerized reminders have increased compliance with standards, significantly reduced physician error, and improved health care outcomes [15,16]. Our findings suggest that the number of immunization visits could be reduced by properly using all visits to administer a necessary vaccine.

Third, physicians and other health professionals may not be paying due attention to recording immunizations. Although it is possible that they do not document vaccinations, this would not explain the large differences between potential and actual immunization. It is also possible that health professionals record immunization, even when no immunizations are given. Regardless, it is important to inform health professionals of adhering to best practices in record-keeping. Monitoring missed opportunities may also be warranted.

Finally, our finding that immunization coverage was lower when facilities did not have a shortage on the day of our facility interview deserves further attention. One would expect more coverage for facilities with no shortage. However, the high coverage among facilities with shortage may indicate that facilities provide the vaccine when available but tend to run out of it. This highlights supply issues rather than errors by the medical team and needs further investigation.

Our finding of low coverage for MMR is Mesoamerica is puzzling. Indeed, with such a coverage one would expect outbreaks in the region. Therefore, it is possible that such outbreaks are not captured or herd immunity in the region may be achieved at a lower rate of coverage. It is more likely that the first is more likely. Hence, our study calls for improving the surveillance systems in place to better detect outbreaks.

The low MMR coverage in Mexico led us to further investigation. In Mexico, the measles and rubella (MR) vaccine is recommended as a booster for children. When we combined MMR and MR, the coverage went up from 44.6% to 75.2%. Days at risk declined from 483 to 274 while potential days at risk declined from 185 to 153. This suggests that physicians provided MR in lieu of MMR when it was unavailable. In fact, 1,430 children received MR and had no record of MMR, although no clear patterns by facility were observed.

Children at risk for measles, mumps, and rubella pose a significant public health problem. Measles is an extremely contagious disease transmitted through respiratory droplets or aerosolized droplet nuclei, and the virus remains infectious within closed spaces for hours [17]. Before the introduction of an effective vaccine in 1963, all children were expected to contract the disease after the first exposure. In 1999, with 90% coverage of one dose of measles-containing vaccine by 35 months of age and the introduction of a routine two-dose schedule before school entry, only 100 cases of measles disease were recorded among children in the United States [18].

Coverage of this nature will increase, and days at risk will decrease, if we avoid missed opportunities and administer vaccines at the required intervals [19]. Our analysis included only vaccination visits; if we used all clinic visits, the potential coverage increases would be higher and the days at risk lower [19]. Also, only children with vaccine cards were included in our measure of potential coverage; those without vaccine cards likely are unvaccinated.

Our findings are the first representative data available on missed opportunities for vaccination in poor areas in Mesoamerica. SM2015 data and this analysis provide unique population-based estimates of vaccination coverage against which prevention efforts may be evaluated.

As we confront the challenge of improving vaccination coverage, it is important to use all health visits to vaccinate or promote vaccination. Vaccination coverage for all antigens will increase by avoiding missed opportunities. For coverage to increase and for elimination to be achieved, a wide range of participants have to be involved, including parents, nurses, pediatricians, and other health providers. Development and implementation of effective public health strategies to limit missed opportunities for vaccination are urgently needed.

## Supporting Information

S1 Table Vaccination schedule by country. (DOCX)

S2 Table Total number of vaccines received at missed opportunity visit. (XLSX)

S3 Table Proportion of children who received an antigen, among children with a missed opportunity (XLSX)

## Abbreviations

MMR: measles, mumps, rubella
SM2015: Salud Mesoamérica 2015
IHME: Institute for Health Metrics and Evaluation
ORS: oral rehydration salts
OR: odds ratio
CI: confidence interval
MR: measles and rubella

## References

[1] Centers for Disease Control and Prevention. Global measles mortality, 2000–2008. Morb Mortal Wkly Rep 2009; 58: 1321–6. 19959985

[2] Global control and regional elimination of measles, 2000–2012. Relevé Épidémiologique Hebd Sect Hygiène Secrétariat Société Nations Wkly Epidemiol Rec Health Sect Secr Leag Nations 2014; 89: 45–52. 24524163

[3] Strebel PM, Cochi SL, Hoekstra E, Rota PA, Feathersone D, Bellini WJ, et al A World Without Measles. J Infect Dis 2011; 204: S1–S3. 10.1093/infdis/jir111 21666150

[4] Ten Great Public Health Achievements—Worldwide, 2001–2010. http://www.cdc.gov/mmwr/preview/mmwrhtml/mm6024a4.htm (accessed Feb 9, 2015).

[5] McCarthy M. Measles outbreak linked to Disney theme parks reaches five states and Mexico. BMJ 2015; 350: h436–h436. 25616478 10.1136/bmj.h436

[6] M G. The beginning of the end of measles and rubella. JAMA Pediatr 2014; 168: 108–9. 24310954 10.1001/jamapediatrics.2013.4603

[7] Mokdad AH, Colson KE, Zúñiga-Brenes P, Ríos-Zertuche D, Palmisano EB, Alfaro-Porras E, et al Salud Mesoamérica 2015 Initiative: Design, Implementation, and Baseline Findings. Popul Health Metr. 10.1186/s12963-015-0034-4 PMC4327957 25685074

[8] Cox DR. Regression Models and Life-Tables. J R Stat Soc Ser B Methodol 1972; 34: 187–220.

[9] Standards for pediatric immunization practices. Ad Hoc Working Group for the Development of Standards for Pediatric Immunization Practices. JAMA 1993; 269: 1817–22. 8459514

[10] Wood D, Schuster M, Donald-Sherbourne C, Duan N, Mazel R, Halfon N. Reducing missed opportunities to vaccinate during child health visits. How effective are parent education and case management? Arch Pediatr Adolesc Med 1998; 152: 238–43. 9529460 10.1001/archpedi.152.3.238

[11] Wood D, Halfon N, Pereyra M, Hamlin JS, Grabowsky M. Knowledge of the childhood immunization schedule and of contraindications to vaccinate by private and public providers in Los Angeles. Pediatr Infect Dis J 1996; 15: 140–5. 8822287 10.1097/00006454-199602000-00010

[12] Siegel RM, Schubert CJ. Physician beliefs and knowledge about vaccinations. Are Cincinnati doctors giving their best shot? Clin Pediatr (Phila) 1996; 35: 79–83. 8775480 10.1177/000992289603500205

[13] McConnochie KM, Roghmann KJ. Immunization opportunities missed among urban poor children. Pediatrics 1992; 89: 1019–26. 1594341

[14] Szilagyi PG, Rodewald LE, Humiston SG, Pollard L, Klossner K, Jones AM, et al Reducing missed opportunities for immunizations. Easier said than done. Arch Pediatr Adolesc Med 1996; 150: 1193–200. 8904862 10.1001/archpedi.1996.02170360083014

[15] McDonald CJ, Hui SL, Smith DM, Tierney WM, Cohen SJ, Weinberger M, et al Reminders to physicians from an introspective computer medical record. A two-year randomized trial. Ann Intern Med 1984; 100: 130–8. 6691639 10.7326/0003-4819-100-1-130

[16] McDonald CJ. Protocol-based computer reminders, the quality of care and the non-perfectability of man. N Engl J Med 1976; 295: 1351–5. 988482 10.1056/NEJM197612092952405

[17] Bloch AB, Orenstein WA, Ewing WM, Spain WH, Mallison GF, Herrmann KL, et al Measles outbreak in a pediatric practice: airborne transmission in an office setting. Pediatrics 1985; 75: 676–83. 3982900

[18] Centers for Disease Control and Prevention. Impact of vaccines universally recommended for children—United States, 1990–1998. Morb Mortal Wkly Rep 1999; 48: 243–8. 10220251

[19] Kahn JG, Mokdad AH, Deming MS, Roungou JB, Boby AM, Excler JL, et al Avoiding missed opportunities for immunization in the Central African Republic: potential impact on vaccination coverage. Bull World Health Organ 1995; 73: 47–55. 7704925 PMC2486578

