# Supplementary material for: Missed Opportunities for Measles, Mumps, and Rubella (MMR) Immunization in Mesoamerica: Potential Impact on Coverage and Days at Risk
Source: PLoS One. 2015 Oct 27;10(10):e0139680. doi: 10.1371/journal.pone.0139680 (PMC4624243; doi:10.1371/journal.pone.0139680)
Supplement: S1 Table — (DOCX) [file pone.0139680.s001.docx]

S1 Table: Vaccination schedule by country

| **Vaccine** | **Guatemala** | **Honduras** | **Mexico** | **Nicaragua** | **Panama** | **El Salvador** |
| --- | --- | --- | --- | --- | --- | --- |
| BCG | At birth | At birth | At birth | At birth | At birth | At birth |
| Hepatitis B | At birth | At birth | At birth  6-8 weeks  5 months  If not given at birth:  2 months  4 months  6 months |  | At birth |  |
| Pentavalent | 2 months  4 months  6 months | 2 months  4 months  6 months | 2 months  4 months  6 months  18 months | 2 months  4 months  6 months | 2 months  4 months  6 months | 2 months  4 months  6 months  15-18 months |
| Rotavirus | 2 months  4 months | 2 months  4 months | 2 months  4 months | 2 months  4 months  6 months  18 months | 2 doses before age 6 months | 2 months  4 months |
| Pneumo-coccal |  | 2 months  4 months  6 months | 2 months  4 months  12 months | 2 months  4 months  6 months  *Restricted to those born 2012 or later* | 2 months  4 months  6 months  12-15 months | 2 months  4 months  12 months |
| MMR | 1 year | 1 year  Another before age 5 years* | 1 year | 1 year | 1 year  Another before age 5 years* | 1 year  4 years |
| Polio (OPV) | 2 months  4 months  6 months  18 months  48 months | 2 months  4 months  6 months  18 months | Campaign | 2 months  4 months  6 months | 2 months  4 months  6 months  18 months  Between 4-5 years* | 2 months  4 months  6 months  15-18 months  48 months |
| DPT | 18 months  48 months | 18 months  4 years  Must be after 3 pentavalent doses at ages 2, 4, 6 months |  | 18 months (must also be compliant with pentavalent) | Between 4-5 years* | 2 months  4 months  6 months  48 months |
| Tetravalent |  |  |  |  | 18 months |  |
| Yellow fever |  |  |  |  | 12 months |  |
| Hepatitis A |  |  |  |  | 12 months  18 months |  |
| Influenza | Seasonal | Seasonal | Seasonal | Seasonal | 2 doses between 6-11 months  2 additional doses before age 5 years* | Seasonal |

*Measurement is censored for children 0-59 months, so compliance cannot be measured from the data.
